# Disodium (2*RS*,3*SR*)-tartrate

**DOI:** 10.1107/S1600536809037155

**Published:** 2009-09-19

**Authors:** William Arbuckle, Stuart Cartner, Alan R. Kennedy, Catriona A. Morrison

**Affiliations:** a Schering-Plough Research Institute, Newhouse, Motherwell ML1 5SH, Scotland; b Department of Pure and Applied Chemistry, University of Strathclyde, 295 Cathedral Street, Glasgow G1 1XL, Scotland

## Abstract

The asymmetric unit of the anhydrous title compound, 2Na^+^·C_4_H_4_O_6_^2−^, contains two sodium cations and one tartrate anion. Each sodium ion is six coordinate, with bonding to six O atoms from both the carboxyl­ate and hydroxyl groups of the anion. A three-dimensional coordination network is formed with sodium ions stacking in layers along the *c*-axis direction. This network is supported by additional O—H⋯O hydrogen bonds.

## Related literature

For the preparation and structure of the equivalent anhydrous *meso*-tartrate salt, see: Blankensteyn & Kroon (1985 ▶). For similar hydrated tartrate salt examples using sodium or mixed sodium with lithium, potassium, rubidium or ammonium cations, see: Ambady & Kartha (1968 ▶); Suzuki *et al.* (1996 ▶); Buschmann & Luger (1985 ▶); Görbitz & Sagstuen (2008 ▶); Hinazumi & Mitsui (1972 ▶). For the use of tartrates as food additives, see: Vickers *et al.* (2007 ▶).
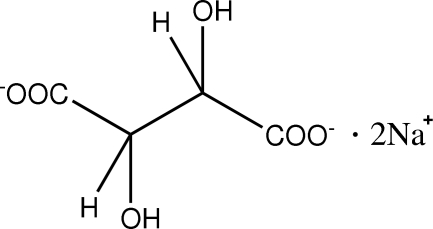


## Experimental

### 

#### Crystal data

2Na^+^·C_4_H_4_O_6_^2−^
*M* *_r_* = 194.06
Orthorhombic, 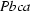

*a* = 10.1160 (4) Å
*b* = 10.0049 (5) Å
*c* = 13.0821 (5) Å
*V* = 1324.03 (10) Å^3^
*Z* = 8
Mo *K*α radiation
μ = 0.29 mm^−1^
*T* = 123 K
0.24 × 0.15 × 0.09 mm

#### Data collection

Oxford Diffraction Gemini S CCD diffractometer
Absorption correction: multi-scan (ABSPACK; Oxford Diffraction, 2007 ▶) *T*_min_ = 0.894, *T*_max_ = 1.000
7156 measured reflections
1934 independent reflections
1566 reflections with *I* > 2/s(*I*)
*R*_int_ = 0.024

#### Refinement

*R*[*F* ^2^ > 2σ(*F* ^2^)] = 0.028
*wR*(*F* ^2^) = 0.072
*S* = 1.05
1934 reflections
117 parameters
H atoms treated by a mixture of independent and constrained refinement
Δρ_max_ = 0.39 e Å^−3^
Δρ_min_ = −0.31 e Å^−3^

### 

Data collection: *CrysAlis CCD* (Oxford Diffraction, 2007 ▶); cell refinement: *CrysAlis CCD*; data reduction: *CrysAlis RED* (Oxford Diffraction, 2007 ▶); program(s) used to solve structure: *SHELXS97* (Sheldrick, 2008 ▶); program(s) used to refine structure: *SHELXL97* (Sheldrick, 2008 ▶); molecular graphics: *ORTEP-3* (Farrugia, 1997 ▶); software used to prepare material for publication: *SHELXL97*.

## Supplementary Material

Crystal structure: contains datablocks global, I. DOI: 10.1107/S1600536809037155/tk2539sup1.cif

Structure factors: contains datablocks I. DOI: 10.1107/S1600536809037155/tk2539Isup2.hkl

Additional supplementary materials:  crystallographic information; 3D view; checkCIF report

## Figures and Tables

**Table 1:** Hydrogen-bond geometry (Å, °)

*D*—H⋯*A*	*D*—H	H⋯*A*	*D*⋯*A*	*D*—H⋯*A*
O3—H2⋯O2^i^	0.900 (17)	1.752 (18)	2.6480 (12)	173.2 (17)
O6—H4⋯O5^ii^	0.883 (17)	1.787 (17)	2.6643 (12)	172.0 (18)

Symmetry codes: (i) 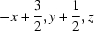; (ii) 
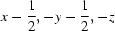
.

**Fig. 1.**
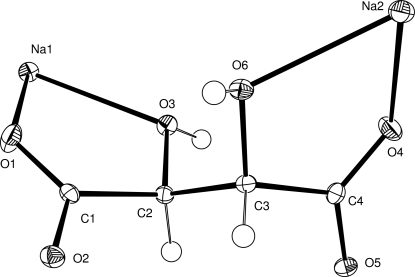
The asymmetric unit and atomic labelling of (I), showing 50% probability displacement ellipsoids. H atoms are shown as spheres of arbitrary radius.

**Fig. 2.**
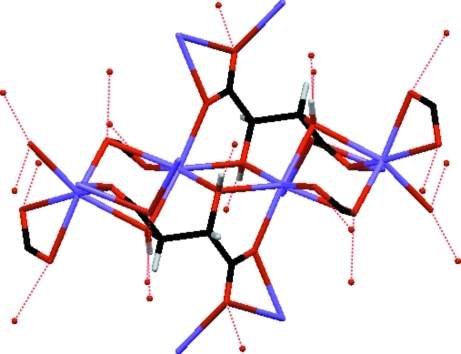
Extended structure formed by bridging O atoms between the Na cations in (I). The Na atoms are purple, O atoms are red, and C atoms are black. Hydrogen bonds are shown as dashed lines.

**Fig. 3.**
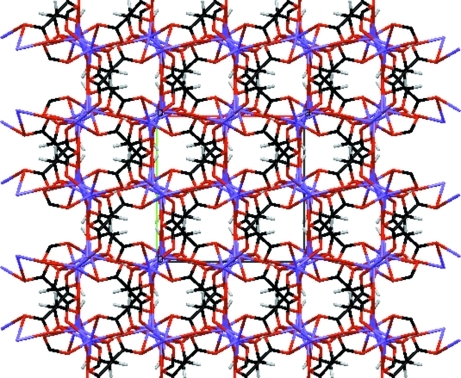
Packed structure of (I) viewed down the c-axis.

## supplementary crystallographic information

### Comment

Tartrate salts are often used as food additives due to their ability to act as anti-oxidants. Disodium tartrate dihydrate, additive number (E335), is used as an emulsifier and binding agent in food products such as jam and sugar syrup (Vickers *et al.*, 2007).

The anhydrous form of racemic disodium tartrate (I) was obtained from aqueous solution. The salt crystallizes in space group Pbca, with two sodium cations and one tartrate anion in the asymmetric unit, Fig. 1. Structures of anhydrous forms of similar materials are uncommon. The extensive structural literature on sodium tartrates and the historically important mixed cation double salts (Na/*X*, with *X* = Li, K, Rb & NH_4_) is dominated by hydrated forms (Ambady & Kartha, 1968; Suzuki *et al.*, 1996; Buschmann & Luger, 1985; Görbitz & Sagstuen, 2008; Hinazumi & Mitsui, 1972). The only other known anhydrous sodium tartrate structure is that of disodium *meso*-tartrate salt (Blankensteyn & Kroon, 1985).

In the present anhydrate, (I), each Na ion forms six bonds to O and each O atom in turn forms two bonds to Na. The range of bond lengths found for Na—O_OOC_ interactions, 2.3097 (10) to 2.5370 (9), encompasses that found for Na—O_OH_ bonds, i.e. 2.3580 (9) to 2.4994 (9) Å. The bond lengths compare well with those observed for disodium D-tartrate dihydrate (Ambady & Kartha, 1968). Each tartrate anion bridges a total of 7 Na ions, see Fig. 2, giving a 3- dimensional coordination network. Figure 3 shows a view of the packed structure, looking down the *c* direction. Note the columns of Na atoms parallel to *c* and also that the apparently empty channels are only 2.5 Å wide and thus are in fact approximate to van der Waals contact distances. This network is supported by intermolecular hydrogen bonding from the OH groups to the carboxylate groups, see Table 1.

### Experimental

Compound (I) was obtained on treating an aqueous solution of (+/-)tartaric acid with an aqueous solution of sodium carbonate. Single-crystals were obtained by allowing the solvent of the reaction mixture to evaporate at 295 K.

### Refinement

Hydroxyl-H atoms were found by difference synthesis and refined isotropically; see Table 1. All other H atoms were positioned geometrically with C—H = 1.00 Å, and with *U*_iso_(H) = 1.2 times *U*_eq_(C).

### Crystal data

**Table:**

2Na^+^·C_4_H_4_O_6_^2^^−^	*F*(000) = 784
*M**_r_* = 194.06	*D*_x_ = 1.947 Mg m^−^^3^
Orthorhombic, *P**b**c**a*	Mo *K*α radiation, λ = 0.71073 Å
Hall symbol: -P 2ac 2ab	Cell parameters from 3917 reflections
*a* = 10.1160 (4) Å	θ = 2.5–30.8°
*b* = 10.0049 (5) Å	µ = 0.29 mm^−^^1^
*c* = 13.0821 (5) Å	*T* = 123 K
*V* = 1324.03 (10) Å^3^	Block, colourless
*Z* = 8	0.24 × 0.15 × 0.09 mm

### Data collection

**Table:**

Oxford Diffraction Gemini S CCD diffractometer	1934 independent reflections
Radiation source: fine-focus sealed tube	1566 reflections with *I* > 2/s(*I*)
graphite	*R*_int_ = 0.024
ω scans	θ_max_ = 30.8°, θ_min_ = 3.1°
Absorption correction: multi-scan (ABSPACK; Oxford Diffraction, 2007)	*h* = −14→8
*T*_min_ = 0.894, *T*_max_ = 1.000	*k* = −14→14
7156 measured reflections	*l* = −17→18

### Refinement

**Table:**

Refinement on *F*^2^	Primary atom site location: structure-invariant direct methods
Least-squares matrix: full	Secondary atom site location: difference Fourier map
*R*[*F*^2^ > 2σ(*F*^2^)] = 0.028	Hydrogen site location: inferred from neighbouring sites
*wR*(*F*^2^) = 0.072	H atoms treated by a mixture of independent and constrained refinement
*S* = 1.05	*w* = 1/[σ^2^(*F*_o_^2^) + (0.0412*P*)^2^] where *P* = (*F*_o_^2^ + 2*F*_c_^2^)/3
1934 reflections	(Δ/σ)_max_ = 0.001
117 parameters	Δρ_max_ = 0.39 e Å^−^^3^
0 restraints	Δρ_min_ = −0.31 e Å^−^^3^

### Special details

**Table:**

Geometry. All e.s.d.'s (except the e.s.d. in the dihedral angle between two l.s. planes) are estimated using the full covariance matrix. The cell e.s.d.'s are taken into account individually in the estimation of e.s.d.'s in distances, angles and torsion angles; correlations between e.s.d.'s in cell parameters are only used when they are defined by crystal symmetry. An approximate (isotropic) treatment of cell e.s.d.'s is used for estimating e.s.d.'s involving l.s. planes.
Refinement. Refinement of *F*^2^ against ALL reflections. The weighted *R*-factor *wR* and goodness of fit *S* are based on *F*^2^, conventional *R*-factors *R* are based on *F*, with *F* set to zero for negative *F*^2^. The threshold expression of *F*^2^ > σ(*F*^2^) is used only for calculating *R*-factors(gt) *etc*. and is not relevant to the choice of reflections for refinement. *R*-factors based on *F*^2^ are statistically about twice as large as those based on *F*, and *R*- factors based on ALL data will be even larger.

### Fractional atomic coordinates and isotropic or equivalent isotropic displacement parameters (Å^2^)

**Table:**

	*x*	*y*	*z*	*U*_iso_*/*U*_eq_	
Na1	0.55185 (4)	−0.03963 (5)	−0.37266 (3)	0.01008 (12)	
Na2	0.52569 (5)	−0.03323 (5)	0.12690 (3)	0.01163 (12)	
O1	0.53147 (9)	−0.24500 (8)	−0.29356 (7)	0.0149 (2)	
O2	0.61519 (8)	−0.40740 (8)	−0.19771 (6)	0.01028 (18)	
O3	0.66678 (8)	−0.05364 (8)	−0.20387 (6)	0.00958 (17)	
O4	0.73339 (8)	−0.01137 (9)	0.05789 (6)	0.01434 (19)	
O5	0.89689 (8)	−0.12972 (8)	−0.01024 (6)	0.01052 (18)	
O6	0.54400 (8)	−0.15557 (9)	−0.02943 (6)	0.00950 (17)	
C1	0.60624 (11)	−0.28543 (11)	−0.22473 (8)	0.0082 (2)	
C2	0.69680 (11)	−0.18581 (11)	−0.16939 (8)	0.0078 (2)	
H1	0.7910	−0.2069	−0.1859	0.009*	
C3	0.67622 (11)	−0.19485 (12)	−0.05305 (8)	0.0082 (2)	
H3	0.6900	−0.2894	−0.0306	0.010*	
C4	0.77515 (10)	−0.10399 (12)	0.00307 (8)	0.0087 (2)	
H2	0.7378 (17)	0.0000 (17)	−0.1980 (13)	0.033 (5)*	
H4	0.4958 (17)	−0.2286 (16)	−0.0224 (14)	0.026 (5)*	

### Atomic displacement parameters (Å^2^)

**Table:**

	*U*^11^	*U*^22^	*U*^33^	*U*^12^	*U*^13^	*U*^23^
Na1	0.0103 (2)	0.0103 (2)	0.0096 (2)	−0.00008 (18)	0.00033 (16)	0.00102 (17)
Na2	0.0123 (2)	0.0126 (2)	0.0100 (2)	0.0033 (2)	0.00156 (16)	0.00156 (17)
O1	0.0174 (4)	0.0114 (4)	0.0158 (4)	−0.0023 (4)	−0.0085 (3)	0.0021 (3)
O2	0.0111 (4)	0.0073 (4)	0.0124 (4)	0.0000 (3)	0.0002 (3)	0.0005 (3)
O3	0.0109 (4)	0.0069 (4)	0.0109 (4)	−0.0012 (3)	−0.0008 (3)	0.0019 (3)
O4	0.0116 (4)	0.0154 (4)	0.0161 (4)	−0.0004 (4)	0.0013 (3)	−0.0077 (3)
O5	0.0078 (3)	0.0112 (4)	0.0125 (4)	0.0004 (3)	0.0002 (3)	0.0009 (3)
O6	0.0074 (4)	0.0097 (4)	0.0114 (4)	−0.0004 (3)	0.0017 (3)	0.0003 (3)
C1	0.0085 (5)	0.0086 (5)	0.0075 (4)	0.0003 (4)	0.0019 (4)	−0.0012 (4)
C2	0.0087 (5)	0.0066 (5)	0.0082 (4)	0.0002 (4)	−0.0003 (4)	0.0002 (4)
C3	0.0073 (5)	0.0081 (5)	0.0092 (4)	0.0009 (4)	0.0000 (4)	0.0001 (4)
C4	0.0100 (5)	0.0092 (5)	0.0069 (5)	−0.0004 (4)	−0.0004 (4)	0.0020 (4)

### Geometric parameters (Å, °)

**Table:**

Na1—O1	2.3097 (10)	O2—C1	1.2737 (14)
Na1—O2^i^	2.3352 (9)	O2—Na1^viii^	2.3352 (9)
Na1—O5^ii^	2.3699 (9)	O2—Na2^vii^	2.5370 (9)
Na1—O4^iii^	2.4095 (9)	O3—C2	1.4298 (14)
Na1—O3	2.4994 (9)	O3—Na2^iv^	2.3580 (9)
Na1—O5^iii^	2.5257 (9)	O3—H2	0.900 (17)
Na1—C4^iii^	2.7875 (12)	O4—C4	1.2457 (13)
Na1—Na2^iv^	3.3886 (6)	O4—Na1^ix^	2.4095 (9)
Na1—Na1^v^	3.5820 (8)	O5—C4	1.2701 (13)
Na2—O4	2.2973 (9)	O5—Na1^x^	2.3699 (9)
Na2—O3^iv^	2.3580 (9)	O5—Na1^ix^	2.5257 (9)
Na2—O6^iv^	2.3856 (9)	O6—C3	1.4279 (13)
Na2—O6	2.3907 (9)	O6—Na2^iv^	2.3856 (9)
Na2—O1^vi^	2.4513 (10)	O6—H4	0.883 (17)
Na2—O2^vi^	2.5370 (9)	C1—C2	1.5352 (16)
Na2—C1^vi^	2.7790 (12)	C1—Na2^vii^	2.7790 (12)
Na2—C4	3.0813 (12)	C2—C3	1.5389 (14)
Na2—Na1^iv^	3.3886 (6)	C2—H1	1.0000
Na2—Na2^iv^	3.4259 (9)	C3—C4	1.5385 (15)
O1—C1	1.2435 (13)	C3—H3	1.0000
O1—Na2^vii^	2.4513 (10)	C4—Na1^ix^	2.7875 (12)
			
O1—Na1—O2^i^	105.24 (3)	O6—Na2—Na1^iv^	160.01 (3)
O1—Na1—O5^ii^	83.81 (3)	O1^vi^—Na2—Na1^iv^	78.32 (2)
O2^i^—Na1—O5^ii^	89.52 (3)	O2^vi^—Na2—Na1^iv^	43.53 (2)
O1—Na1—O4^iii^	115.96 (4)	C1^vi^—Na2—Na1^iv^	62.98 (3)
O2^i^—Na1—O4^iii^	132.93 (4)	C4—Na2—Na1^iv^	137.55 (3)
O5^ii^—Na1—O4^iii^	115.67 (3)	O4—Na2—Na2^iv^	74.89 (3)
O1—Na1—O3	66.16 (3)	O3^iv^—Na2—Na2^iv^	102.54 (3)
O2^i^—Na1—O3	91.15 (3)	O6^iv^—Na2—Na2^iv^	44.23 (2)
O5^ii^—Na1—O3	149.03 (3)	O6—Na2—Na2^iv^	44.11 (2)
O4^iii^—Na1—O3	85.73 (3)	O1^vi^—Na2—Na2^iv^	126.20 (3)
O1—Na1—O5^iii^	159.12 (4)	O2^vi^—Na2—Na2^iv^	167.44 (3)
O2^i^—Na1—O5^iii^	92.83 (3)	C1^vi^—Na2—Na2^iv^	148.01 (3)
O5^ii^—Na1—O5^iii^	86.00 (3)	C4—Na2—Na2^iv^	70.08 (2)
O4^iii^—Na1—O5^iii^	53.42 (3)	Na1^iv^—Na2—Na2^iv^	147.42 (2)
O3—Na1—O5^iii^	124.87 (3)	C1—O1—Na1	124.02 (7)
O1—Na1—C4^iii^	140.88 (4)	C1—O1—Na2^vii^	91.57 (7)
O2^i^—Na1—C4^iii^	113.09 (4)	Na1—O1—Na2^vii^	128.11 (4)
O5^ii^—Na1—C4^iii^	103.55 (3)	C1—O2—Na1^viii^	126.92 (7)
O4^iii^—Na1—C4^iii^	26.47 (3)	C1—O2—Na2^vii^	87.02 (6)
O3—Na1—C4^iii^	104.60 (3)	Na1^viii^—O2—Na2^vii^	88.03 (3)
O5^iii^—Na1—C4^iii^	27.09 (3)	C2—O3—Na2^iv^	112.43 (6)
O1—Na1—Na2^iv^	75.27 (3)	C2—O3—Na1	115.43 (6)
O2^i^—Na1—Na2^iv^	48.44 (2)	Na2^iv^—O3—Na1	88.42 (3)
O5^ii^—Na1—Na2^iv^	122.82 (3)	C2—O3—H2	110.8 (11)
O4^iii^—Na1—Na2^iv^	121.39 (3)	Na2^iv^—O3—H2	113.8 (11)
O3—Na1—Na2^iv^	44.07 (2)	Na1—O3—H2	114.4 (11)
O5^iii^—Na1—Na2^iv^	125.40 (3)	C4—O4—Na2	117.75 (7)
C4^iii^—Na1—Na2^iv^	126.00 (3)	C4—O4—Na1^ix^	93.95 (7)
O1—Na1—Na1^v^	125.98 (3)	Na2—O4—Na1^ix^	134.16 (4)
O2^i^—Na1—Na1^v^	91.68 (3)	C4—O5—Na1^x^	130.76 (7)
O5^ii^—Na1—Na1^v^	44.70 (2)	C4—O5—Na1^ix^	88.02 (7)
O4^iii^—Na1—Na1^v^	82.32 (2)	Na1^x^—O5—Na1^ix^	94.00 (3)
O3—Na1—Na1^v^	166.03 (3)	C3—O6—Na2^iv^	112.27 (6)
O5^iii^—Na1—Na1^v^	41.30 (2)	C3—O6—Na2	113.49 (6)
C4^iii^—Na1—Na1^v^	61.79 (2)	Na2^iv^—O6—Na2	91.66 (3)
Na2^iv^—Na1—Na1^v^	140.09 (2)	C3—O6—H4	108.1 (11)
O4—Na2—O3^iv^	152.61 (4)	Na2^iv^—O6—H4	123.2 (11)
O4—Na2—O6^iv^	89.13 (3)	Na2—O6—H4	107.0 (12)
O3^iv^—Na2—O6^iv^	72.09 (3)	O1—C1—O2	123.77 (10)
O4—Na2—O6	69.00 (3)	O1—C1—C2	119.55 (10)
O3^iv^—Na2—O6	128.17 (3)	O2—C1—C2	116.66 (9)
O6^iv^—Na2—O6	88.34 (3)	O1—C1—Na2^vii^	61.86 (6)
O4—Na2—O1^vi^	103.37 (3)	O2—C1—Na2^vii^	65.74 (6)
O3^iv^—Na2—O1^vi^	99.91 (3)	C2—C1—Na2^vii^	158.15 (7)
O6^iv^—Na2—O1^vi^	161.92 (4)	O3—C2—C1	108.96 (9)
O6—Na2—O1^vi^	84.14 (3)	O3—C2—C3	109.73 (9)
O4—Na2—O2^vi^	92.94 (3)	C1—C2—C3	110.33 (9)
O3^iv^—Na2—O2^vi^	89.70 (3)	O3—C2—H1	109.3
O6^iv^—Na2—O2^vi^	140.73 (3)	C1—C2—H1	109.3
O6—Na2—O2^vi^	128.77 (3)	C3—C2—H1	109.3
O1^vi^—Na2—O2^vi^	52.82 (3)	O6—C3—C4	110.09 (9)
O4—Na2—C1^vi^	93.93 (3)	O6—C3—C2	108.94 (8)
O3^iv^—Na2—C1^vi^	100.63 (3)	C4—C3—C2	110.44 (9)
O6^iv^—Na2—C1^vi^	167.66 (4)	O6—C3—H3	109.1
O6—Na2—C1^vi^	103.92 (4)	C4—C3—H3	109.1
O1^vi^—Na2—C1^vi^	26.57 (3)	C2—C3—H3	109.1
O2^vi^—Na2—C1^vi^	27.24 (3)	O4—C4—O5	123.96 (10)
O4—Na2—C4	20.96 (3)	O4—C4—C3	119.58 (10)
O3^iv^—Na2—C4	170.22 (3)	O5—C4—C3	116.46 (10)
O6^iv^—Na2—C4	98.22 (3)	O4—C4—Na1^ix^	59.58 (6)
O6—Na2—C4	50.89 (3)	O5—C4—Na1^ix^	64.89 (6)
O1^vi^—Na2—C4	89.75 (3)	C3—C4—Na1^ix^	172.43 (7)
O2^vi^—Na2—C4	97.44 (3)	O4—C4—Na2	41.28 (5)
C1^vi^—Na2—C4	88.69 (3)	O5—C4—Na2	155.89 (7)
O4—Na2—Na1^iv^	124.34 (3)	C3—C4—Na2	81.60 (6)
O3^iv^—Na2—Na1^iv^	47.50 (2)	Na1^ix^—C4—Na2	95.11 (3)
O6^iv^—Na2—Na1^iv^	105.57 (3)		
			
O2^i^—Na1—O1—C1	−103.88 (9)	Na2^iv^—O3—C2—C1	78.38 (8)
O5^ii^—Na1—O1—C1	168.31 (9)	Na1—O3—C2—C1	−21.02 (10)
O4^iii^—Na1—O1—C1	52.72 (10)	Na2^iv^—O3—C2—C3	−42.52 (10)
O3—Na1—O1—C1	−19.48 (9)	Na1—O3—C2—C3	−141.92 (7)
O5^iii^—Na1—O1—C1	107.05 (12)	O1—C1—C2—O3	5.65 (14)
C4^iii^—Na1—O1—C1	64.41 (11)	O2—C1—C2—O3	−175.83 (9)
Na2^iv^—Na1—O1—C1	−65.38 (9)	Na2^vii^—C1—C2—O3	93.2 (2)
Na1^v^—Na1—O1—C1	152.51 (8)	O1—C1—C2—C3	126.19 (11)
O2^i^—Na1—O1—Na2^vii^	131.26 (5)	O2—C1—C2—C3	−55.30 (13)
O5^ii^—Na1—O1—Na2^vii^	43.45 (5)	Na2^vii^—C1—C2—C3	−146.27 (17)
O4^iii^—Na1—O1—Na2^vii^	−72.13 (6)	Na2^iv^—O6—C3—C4	80.01 (8)
O3—Na1—O1—Na2^vii^	−144.34 (6)	Na2—O6—C3—C4	−22.24 (10)
O5^iii^—Na1—O1—Na2^vii^	−17.81 (13)	Na2^iv^—O6—C3—C2	−41.23 (10)
C4^iii^—Na1—O1—Na2^vii^	−60.45 (7)	Na2—O6—C3—C2	−143.48 (7)
Na2^iv^—Na1—O1—Na2^vii^	169.76 (5)	O3—C2—C3—O6	56.23 (12)
Na1^v^—Na1—O1—Na2^vii^	27.65 (7)	C1—C2—C3—O6	−63.83 (12)
O1—Na1—O3—C2	20.93 (7)	O3—C2—C3—C4	−64.79 (11)
O2^i^—Na1—O3—C2	127.10 (7)	C1—C2—C3—C4	175.14 (9)
O5^ii^—Na1—O3—C2	36.12 (11)	Na2—O4—C4—O5	−154.27 (9)
O4^iii^—Na1—O3—C2	−99.92 (7)	Na1^ix^—O4—C4—O5	−8.65 (11)
O5^iii^—Na1—O3—C2	−138.64 (7)	Na2—O4—C4—C3	25.69 (12)
C4^iii^—Na1—O3—C2	−118.66 (7)	Na1^ix^—O4—C4—C3	171.31 (8)
Na2^iv^—Na1—O3—C2	114.18 (8)	Na2—O4—C4—Na1^ix^	−145.62 (7)
Na1^v^—Na1—O3—C2	−131.23 (13)	Na1^ix^—O4—C4—Na2	145.62 (7)
O1—Na1—O3—Na2^iv^	−93.25 (4)	Na1^x^—O5—C4—O4	−85.34 (13)
O2^i^—Na1—O3—Na2^iv^	12.92 (3)	Na1^ix^—O5—C4—O4	8.23 (11)
O5^ii^—Na1—O3—Na2^iv^	−78.06 (7)	Na1^x^—O5—C4—C3	94.70 (11)
O4^iii^—Na1—O3—Na2^iv^	145.90 (3)	Na1^ix^—O5—C4—C3	−171.72 (8)
O5^iii^—Na1—O3—Na2^iv^	107.18 (4)	Na1^x^—O5—C4—Na1^ix^	−93.57 (8)
C4^iii^—Na1—O3—Na2^iv^	127.16 (3)	Na1^x^—O5—C4—Na2	−129.86 (16)
Na1^v^—Na1—O3—Na2^iv^	114.59 (13)	Na1^ix^—O5—C4—Na2	−36.3 (2)
O3^iv^—Na2—O4—C4	−161.78 (8)	O6—C3—C4—O4	−1.01 (13)
O6^iv^—Na2—O4—C4	−116.05 (8)	C2—C3—C4—O4	119.33 (11)
O6—Na2—O4—C4	−27.50 (7)	O6—C3—C4—O5	178.95 (9)
O1^vi^—Na2—O4—C4	50.75 (8)	C2—C3—C4—O5	−60.71 (13)
O2^vi^—Na2—O4—C4	103.18 (8)	O6—C3—C4—Na1^ix^	80.5 (6)
C1^vi^—Na2—O4—C4	75.91 (8)	C2—C3—C4—Na1^ix^	−159.2 (5)
Na1^iv^—Na2—O4—C4	135.61 (7)	O6—C3—C4—Na2	15.80 (7)
Na2^iv^—Na2—O4—C4	−73.63 (7)	C2—C3—C4—Na2	136.14 (8)
O3^iv^—Na2—O4—Na1^ix^	69.96 (10)	O3^iv^—Na2—C4—O4	57.9 (2)
O6^iv^—Na2—O4—Na1^ix^	115.68 (6)	O6^iv^—Na2—C4—O4	65.18 (8)
O6—Na2—O4—Na1^ix^	−155.76 (6)	O6—Na2—C4—O4	146.25 (9)
O1^vi^—Na2—O4—Na1^ix^	−77.51 (6)	O1^vi^—Na2—C4—O4	−131.11 (8)
O2^vi^—Na2—O4—Na1^ix^	−25.08 (6)	O2^vi^—Na2—C4—O4	−78.70 (8)
C1^vi^—Na2—O4—Na1^ix^	−52.35 (6)	C1^vi^—Na2—C4—O4	−104.56 (8)
C4—Na2—O4—Na1^ix^	−128.26 (11)	Na1^iv^—Na2—C4—O4	−58.85 (9)
Na1^iv^—Na2—O4—Na1^ix^	7.34 (7)	Na2^iv^—Na2—C4—O4	99.85 (8)
Na2^iv^—Na2—O4—Na1^ix^	158.11 (6)	O4—Na2—C4—O5	61.8 (2)
O4—Na2—O6—C3	25.51 (7)	O3^iv^—Na2—C4—O5	119.7 (2)
O3^iv^—Na2—O6—C3	−179.25 (7)	O6^iv^—Na2—C4—O5	127.0 (2)
O6^iv^—Na2—O6—C3	115.22 (8)	O6—Na2—C4—O5	−151.9 (2)
O1^vi^—Na2—O6—C3	−81.24 (7)	O1^vi^—Na2—C4—O5	−69.3 (2)
O2^vi^—Na2—O6—C3	−50.74 (9)	O2^vi^—Na2—C4—O5	−16.9 (2)
C1^vi^—Na2—O6—C3	−63.37 (8)	C1^vi^—Na2—C4—O5	−42.7 (2)
C4—Na2—O6—C3	13.22 (7)	Na1^iv^—Na2—C4—O5	3.0 (2)
Na1^iv^—Na2—O6—C3	−109.91 (9)	Na2^iv^—Na2—C4—O5	161.7 (2)
Na2^iv^—Na2—O6—C3	115.22 (8)	O4—Na2—C4—C3	−157.60 (11)
O4—Na2—O6—Na2^iv^	−89.70 (3)	O3^iv^—Na2—C4—C3	−99.7 (2)
O3^iv^—Na2—O6—Na2^iv^	65.53 (4)	O6^iv^—Na2—C4—C3	−92.43 (6)
O6^iv^—Na2—O6—Na2^iv^	0.0	O6—Na2—C4—C3	−11.35 (6)
O1^vi^—Na2—O6—Na2^iv^	163.54 (4)	O1^vi^—Na2—C4—C3	71.29 (6)
O2^vi^—Na2—O6—Na2^iv^	−165.95 (4)	O2^vi^—Na2—C4—C3	123.70 (6)
C1^vi^—Na2—O6—Na2^iv^	−178.59 (4)	C1^vi^—Na2—C4—C3	97.84 (6)
C4—Na2—O6—Na2^iv^	−102.00 (4)	Na1^iv^—Na2—C4—C3	143.55 (5)
Na1^iv^—Na2—O6—Na2^iv^	134.88 (8)	Na2^iv^—Na2—C4—C3	−57.75 (5)
Na1—O1—C1—O2	−163.11 (8)	O4—Na2—C4—Na1^ix^	29.27 (7)
Na2^vii^—O1—C1—O2	−23.34 (11)	O3^iv^—Na2—C4—Na1^ix^	87.1 (2)
Na1—O1—C1—C2	15.30 (14)	O6^iv^—Na2—C4—Na1^ix^	94.44 (4)
Na2^vii^—O1—C1—C2	155.06 (9)	O6—Na2—C4—Na1^ix^	175.52 (5)
Na1—O1—C1—Na2^vii^	−139.77 (8)	O1^vi^—Na2—C4—Na1^ix^	−101.84 (3)
Na1^viii^—O2—C1—O1	−62.75 (14)	O2^vi^—Na2—C4—Na1^ix^	−49.43 (4)
Na2^vii^—O2—C1—O1	22.53 (11)	C1^vi^—Na2—C4—Na1^ix^	−75.29 (4)
Na1^viii^—O2—C1—C2	118.80 (9)	Na1^iv^—Na2—C4—Na1^ix^	−29.59 (5)
Na2^vii^—O2—C1—C2	−155.91 (9)	Na2^iv^—Na2—C4—Na1^ix^	129.12 (3)
Na1^viii^—O2—C1—Na2^vii^	−85.29 (7)		

Symmetry codes: (i) −*x*+1, *y*+1/2, −*z*−1/2; (ii) *x*−1/2, *y*, −*z*−1/2; (iii) −*x*+3/2, −*y*, *z*−1/2; (iv) −*x*+1, −*y*, −*z*; (v) −*x*+1, −*y*, −*z*−1; (vi) *x*, −*y*−1/2, *z*+1/2; (vii) *x*, −*y*−1/2, *z*−1/2; (viii) −*x*+1, *y*−1/2, −*z*−1/2; (ix) −*x*+3/2, −*y*, *z*+1/2; (x) *x*+1/2, *y*, −*z*−1/2.

### Hydrogen-bond geometry (Å, °)

**Table:**

*D*—H···*A*	*D*—H	H···*A*	*D*···*A*	*D*—H···*A*
O3—H2···O2^xi^	0.900 (17)	1.752 (18)	2.6480 (12)	173.2 (17)
O6—H4···O5^xii^	0.883 (17)	1.787 (17)	2.6643 (12)	172.0 (18)

Symmetry codes: (xi) −*x*+3/2, *y*+1/2, *z*; (xii) *x*−1/2, −*y*−1/2, −*z*.

## References

[bb1] Ambady, G. K. & Kartha, G. (1968). *Acta Cryst.* B**24**, 1540–1547.

[bb2] Blankensteyn, A. J. A. R. & Kroon, J. (1985). *Acta Cryst.* C**41**, 182–184.

[bb3] Buschmann, J. & Luger, P. (1985). *Acta Cryst.* C**41**, 206–208.

[bb4] Farrugia, L. J. (1997). *J. Appl. Cryst.* **30**, 565.

[bb5] Görbitz, C. H. & Sagstuen, E. (2008). *Acta Cryst.* E**64**, m507–m508. 10.1107/S1600536808005266 PMC2960997 21201980

[bb6] Hinazumi, H. & Mitsui, T. (1972). *Acta Cryst.* B**28**, 3299–3305.

[bb7] Oxford Diffraction (2007). *CrysAlis CCD, *CrysAlis RED** and *ABSPACK* Oxford Diffraction Ltd, Abingdon, England.

[bb8] Sheldrick, G. M. (2008). *Acta Cryst.* A**64**, 112–122. 10.1107/S0108767307043930 18156677

[bb9] Suzuki, E., Kabasawa, H., Honma, T., Nozaki, R. & Shiozaki, Y. (1996). *Acta Cryst.* B**52**, 976–981.

[bb10] Vickers, P. J., Braybook, J., Lawrence, P. & Gray, K. (2007). *J. Food. Compos. Anal.* **20**, 252–256.

